# Characterization of 3-Dimensional Printing and Casting Materials for use in Computed Tomography and X-ray Imaging Phantoms

**DOI:** 10.6028/jres.125.029

**Published:** 2020-09-15

**Authors:** B. E. Yunker, A. Holmgren, K. F. Stupic, J. L. Wagner, S. Huddle, R. Shandas, R. F. Weir, K. E. Keenan, E. Garboczi, S. E. Russek

**Affiliations:** 1 Physical Measurement Laboratory, National Institute of Standards and Technology, Boulder, CO 80305, USA; 2 2University of Colorado-Denver/Anschutz, Aurora, CO, USA; 3 Material Measurement Laboratory, National Institute of Standards and Technology, Boulder, CO 80305, USA

**Keywords:** 3D printing, computed tomography, CT, medical imaging, phantom, polymer, X-ray

## Abstract

Imaging phantoms are used to calibrate and validate the performance of medical computed tomography (CT) systems. Many new materials developed for three-dimensional (3D) printing processes may be useful in the direct printing or casting of biomimetic and geometrically accurate CT and X-ray phantoms. The X-ray linear attenuation coefficients of polymer samples were measured to discover materials for use as tissue mimics in phantoms. This study included a cohort of polymer compounds that were tested in cured form. The cohort consisted of 101 standardized polymer samples fabricated from: two-part silicones and polyurethanes used in commercial casting processes; one-part optically cured polyurethanes used in 3D printing; and fused deposition thermoplastics used in 3D printing. The testing was performed with a commercial micro-CT imaging system from 40 kVp to 140 kVp. The X-ray linear coefficients of the samples and human tissues were plotted with error bars to allow the reader to identify suitable mimics. The X-ray linear attenuation coefficients of the tested material samples spanned a wide range of values, with a small number of them overlapping established human tissue mimic values. Twenty 3D printer materials and one castable polyurethane tracked nylon and polymethyl methacrylate (PMMA) as established X-ray mimics for fat. Five 3D printer materials tracked water as an established X-ray mimic for muscle.

## 1 Introduction

X-ray computed tomography (CT) imaging is an invaluable medical diagnostic tool, and calibration phantoms are used to assess stability in CT scans over time, as well as establish consistency between manufacturers and models of scanners [1-4]. The fabrication of CT phantoms historically involves the machining and casting of large plastic components using manual and automated milling equipment. This approach involves considerable labor and/or machine time costs and feature detail that is limited to tool size and range of motion. With the development of multiple-material capability, improving accuracy, and decreasing costs of additive manufacturing (three-dimensional [3D] printing) technology, there are opportunities to fabricate highly detailed phantoms for calibration and patient-specific anatomical models for surgical planning and training. The use of 3D printing materials for CT phantoms and CT-compatible devices and fixtures is well documented in literature [5-12].

In previous research, small numbers of two-part silicone and polyurethane polymers were imaged with CT, magnetic resonance imaging (MRI), and ultrasound [13, 14]. The imaging results suggested that some of the materials might be suitable for use in phantoms, and that the viscosities of the uncured polymer components were found to be compatible with 3D printing systems using sub-millimeter-size nozzles.

Medical imaging systems use X-ray tubes for low cost and portability. These tubes emit energy over a wide spectrum of energies (polyenergetic) that vary between manufacturer and over tube life. The beam energy may be selectively filtered (hardened) using metal plates of specific thickness to attenuate unwanted energies [15-17].

Gamma-ray sources, involving the nuclear decay of isotopes such as ^99m^Tc, ^131^I, and ^137^C, are utilized for measuring the linear attenuation coefficients of tissues and materials because their characteristic emissions are contained in narrow (monoenergetic) bands of the energy spectrum [18-23].

This research was performed to discover materials with X-ray (polyenergetic) linear attenuation coefficients similar to human tissues for use as biomimics or materials that provide discernible contrast relative to tissues for use as fiducials or fixturing.

## 2 Methods

A list of the physical properties of candidate materials and reference tissues was compiled from manufacturer data sheets and peer-reviewed journal papers [20-27]. The selection criteria for test sample fabrication included material density, hardness, and elongation characteristics that were similar to human tissue values, in addition to good availability, low cost, easy handling, and low toxicity.

Samples of 3D printing materials were fabricated as cylinders 10 mm in diameter and 10 mm high (± 0.1 mm). Standard samples of one-part ultraviolet-cured polyurethanes were printed with a FormLabs (Somerville, MA) (www.formlabs.com) Form 2 stereolithographic laser (SLA) printer.^1^1 Certain commercial equipment, instruments, and/or materials are identified in this report in order to adequately specify the experimental procedure. Such identification does not imply recommendation or endorsement by the National Institute of Standards and Technology, nor does it imply that the equipment and/or materials used are necessarily the best available for the purpose. Standard samples of one-part polyurethanes and fused deposition modeling (FDM) materials were procured from third-party 3D printing fabricators Protocam (Allentown, PA) (www.protocam.com), Protogenic (Westminster, CO) (www.tenere.com), Protolabs (Maple Plain, MN) (www.protolabs.com), and Sculpteo (San Leandro, CA) (www.sculpteo.com). Since printer manufacturers offer materials optimized for each printer model and offer some compatibility with third-party materials, this sourcing strategy gave access to materials and chemistries from all major suppliers (3Dsystems, ALM, Carbon, Carbon Resin, DSM Somos, EOS, and Stratasys).

Samples of molded silicones were fabricated as cylinders 10 mm in diameter and 10 mm high (± 0.5 mm) from die cuts of precast material obtained from Silicones, Inc. (High Point, NC) (www.silicones-inc.com), and from Smooth-On, Inc. (Macungie, PA) (www.smooth-on.com). A sample of a two-part polyurethane from Huntsman (Woodlands, TX) (www.freeman.com) was also die cut. A borosilicate glass vial with an internal diameter of 10 mm was used to hold the water reference, and the 10 mm diameter aluminum reference was turned down from bar stock.

Linear attenuation was chosen as the measured parameter for two reasons. First, the contrast between two overlaying or adjacent materials is easily computed from the ratios of the material linear attenuation coefficients and their thicknesses. Second, the stated densities of the materials could not be used in mass attenuation calculations because they varied in fabricated form due to random density reduction from microscopic air inclusion in the cast materials and the random inherent inter-filament and intra-filament gaps formed during the deposition of melted 3D printing materials.

The calculation of the linear attenuation coefficient, µ, for the samples is described by Eq. (1) as

$µ\;({cm}^{-1})=\frac{1}{x}\;ln\frac{I0}{Is}$ (1)

where Is = average pixel intensity within the region of interest (ROI) on the material sample; I_0_ = average pixel intensity of the background behind the sample; and *x* = thickness of the sample [20].

**Fig. 1:**
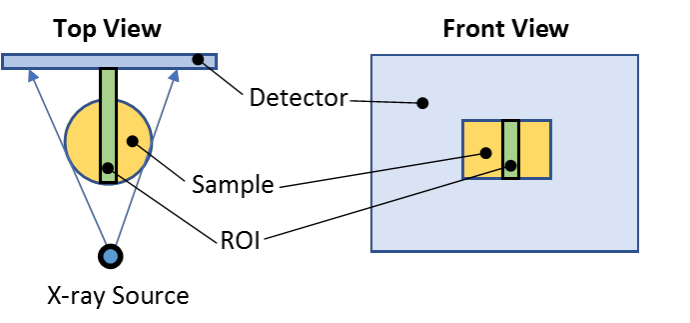
Measurement setup.

The test samples were placed on the imaging platform of a North Star Imaging Systems X-VIEW X50 X-ray micro-CT imaging system, which was maintained and calibrated by the manufacturer under contract. Since the samples were cylindrical and homogeneous, the intensity values of the samples (Is) were captured within a fixed rectangular ROI of 350 pixels (35 pixels vertical × 10 pixels horizontal) centered on the sample within a single two-dimensional (2D) image as shown in Fig. 1.

**Fig. 2:**
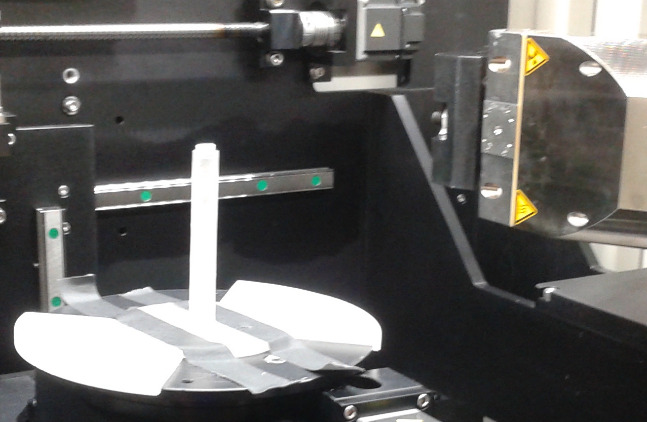
Sample stand with locator wings in micro-CT imaging chamber.

To ensure consistent ROI location on the samples, a sample stand with locating wings was designed to fit over the micro-CT scanner turntable as shown in Fig. 2.

The stand was designed as three pieces to facilitate fabrication on a 3D printer. This setup minimized the effects of sample curvature on the front and back surfaces, allowing the full 10 mm of the sample diameter to be used in the linear attenuation coefficient calculation. The error contribution of the variation in sample diameter was established at ± 5% based on measurements taken on a random group of samples fabricated by both casting and printing methods. This error was added to the ± 3% measurement error of the X-ray system.

The data were taken using an automated script that varied the X-ray tube voltage from 40 kVp to 140 kVp (kilovolts peak) to provide ten data points in 10 kVp linear increments. To maintain constant reference counts and maximum detector dynamic range, the tube current was allowed to vary from 27 µA to 550 µA across the selected voltages. Lower kVp values were not used as they were problematic for the imaging system power supply stability and settling time.

To compensate for the inherent spatial image variations in the scintillator and imaging detector, flat field images were taken before and after the imaging of each group of 20 samples and were used to normalize the sample image data before computing attenuation coefficients.

The sample volume of the micro-CT imaging system was enclosed and employed an interlocked safety door which precluded independent measurement of the energy at the detector.

The calculated linear attenuation coefficients and those from literature were plotted as overlays to the measured/calculated values to allow the reader to identify materials that may be usable for their application after accounting for their own system’s tube efficiency and beam hardening. Some overlay plots include linear coefficients calculated from atomic composition [27]. The attenuation coefficients of the materials imaged with the poly-energetic X-ray beam of the micro-CT system were expected to be higher than the standards values measured with monoenergetic sources since the energy incident at the sample in keV (kilo electron volts) will be less than that applied to the X-ray tube in kVp. To accommodate this expected difference, several materials established by Geraldelli *et al*. as X-ray tissue mimics were included in the testing as relative references, and the resulting plots combine data in kVp and keV [24]. Samples of nylon and polymethyl methacrylate (PMMA) were included in the testing as established X-ray fat mimics. Water was included as an established X-ray mimic for muscle. Aluminum was included as a well-documented reference [19]. A bone reference was not included since the laboratory was not approved for biomaterials.

All plot data were sorted using 40 keV/kVp values to give the legend and lines a top down sort order. All data was also provided in tabular form including curve fit parameters to enable specific material comparisons, and physical characteristics that may be useful to phantom fabrication. The 3D printed samples were printed at maximum printer resolution as listed in the tables along with the manufacturer specified material density.

The International Commission on Radiation Units and Measurements (ICRU) Report 44 recommends that tissue-equivalent materials should have linear attenuation coefficients within ± 5% of a given body tissue over the required photon energy interval for image quality assessments in diagnostic radiology [27]. For the purposes of this research, materials with error bars that overlap those of the literature-reported human tissues could be considered for use as mimics.

## 3 Results

**Fig. A1:**
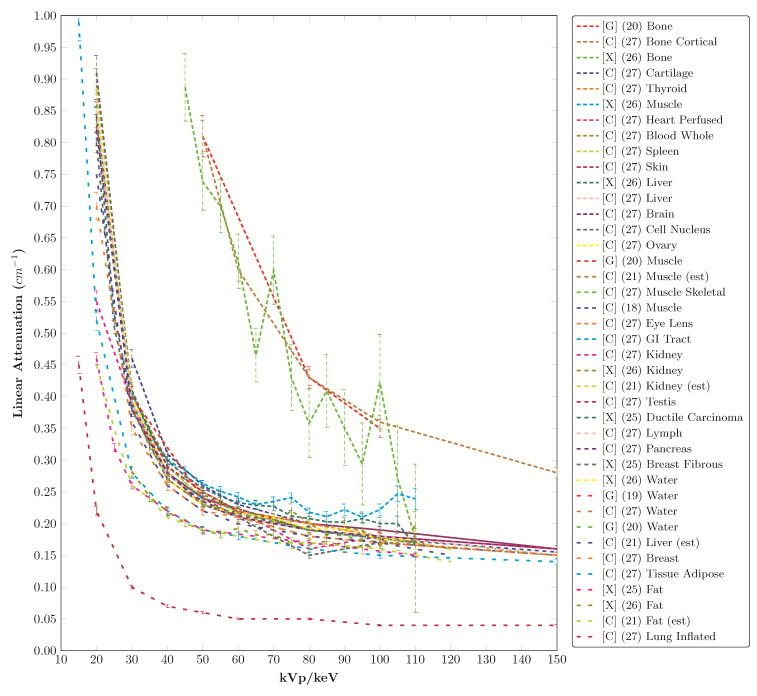
Linear attenuation coefficients for human tissues.

The properties of over 1200 castable, printable, and human tissues were reviewed for candidate materials. The linear attenuation coefficients for tissues found in literature were sorted using 40 kVp/keV attenuation values in Table A1 with the data plotted in Fig. A1. Although the Fig. A1 plots span 10 keV to 150 keV, there were few entries in Table A1 above 110 keV, so those columns were omitted from the tables to minimize journal space.

**Fig. A2:**
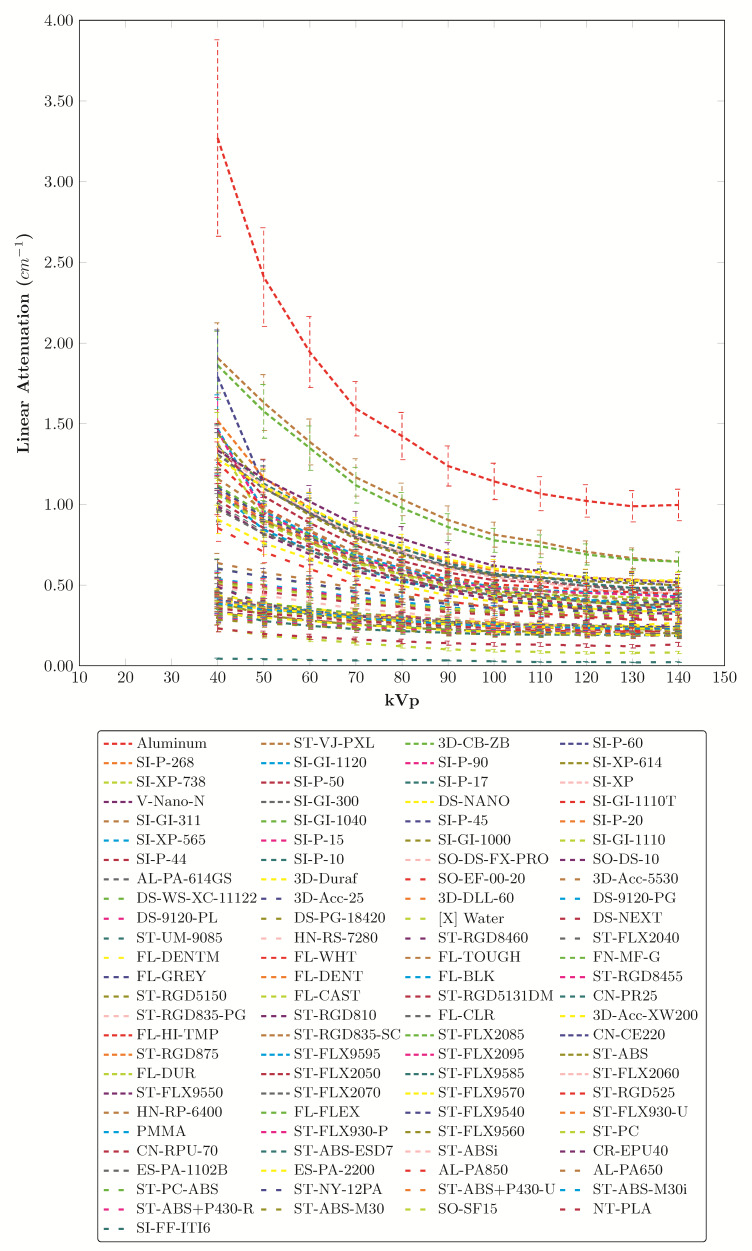
Linear attenuation coefficients for all materials tested in this X-ray source study (legend order is left to right and top to bottom).

The physical characteristics and linear attenuation coefficients of all the tested materials samples are listed in Table A2 with the data plotted in Fig. A2.

**Fig. 3:**
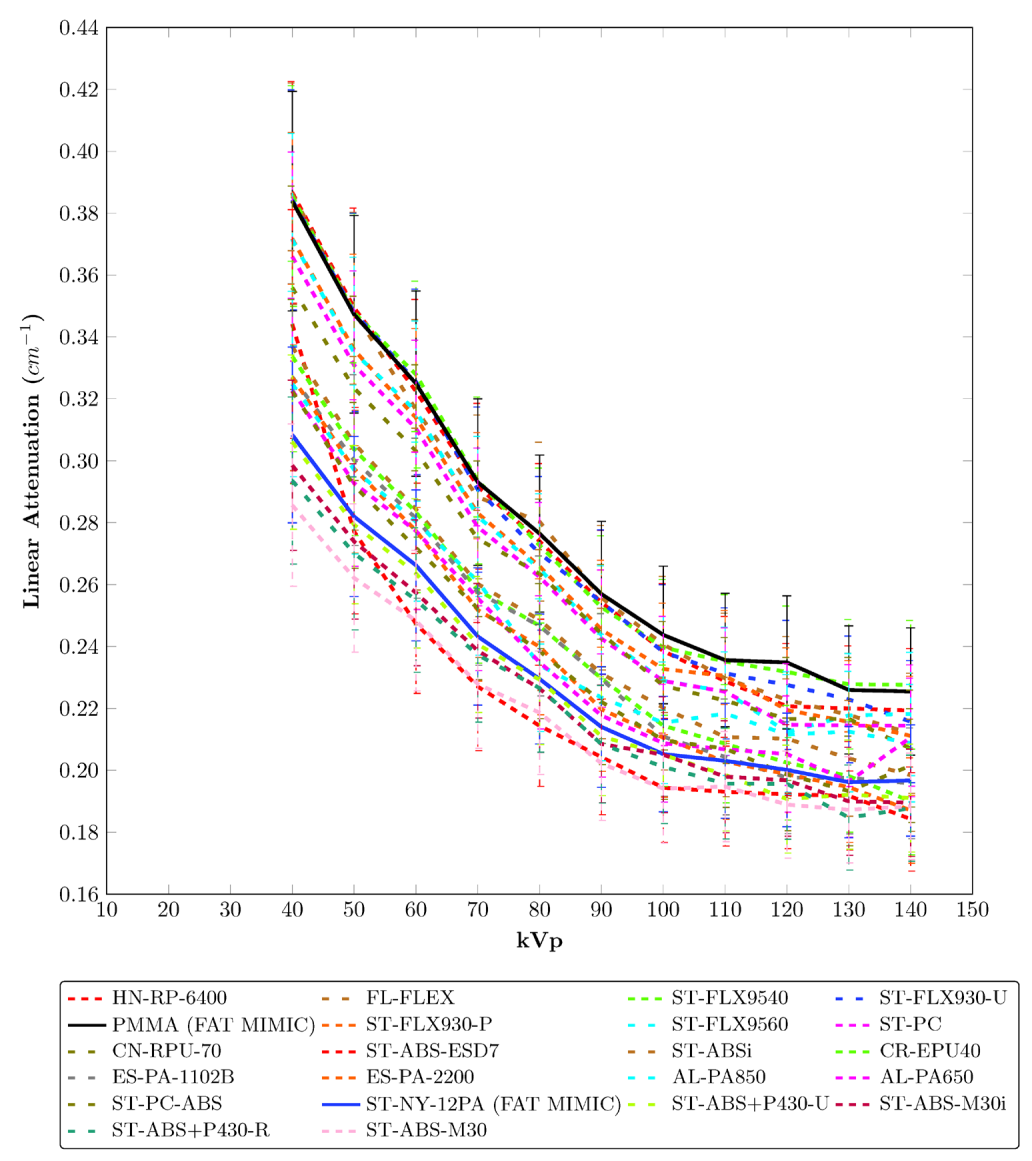
Candidate fat mimics in this study (similar to PMMA and nylon) (legend order is left to right and top to bottom). Average error relative to PMMA (solid black line): HN-RP-6400 1.9%, FL-FLEX 2.3%, ST-FLX9540 0.9%, ST-FLX930-U 1.6%, ST-FLX930-P 4.2%, ST-FLX9560 4.5%, ST-PC 5.3%, and CN-RPU-70 6.3%. Average error relative to nylon, (ST-NY-12PA [solid blue line]): ST-ABS-ESD7 5.5%, ST-ABSi 6.2%, CR-EPU40 5.1%, ES-PA-1102B 4.1%, ES-PA-2200 3.2%, AL-PA850 5.7%, AL-PA650 3.2%, ST-PC-ABS 2.8%, ST-ABS+P430-U 1.6%, ST-ABS-M30i 2.4%, ST-ABS+P430-R 3.4%, and ST-ABS-M30 5. 6%.

The materials from this study with linear attenuation coefficients close to the materials identified in literature as polyenergetic (X-ray source) fat mimics for PMMA and nylon are plotted in Fig. 3 [24-26].

**Fig. 4:**
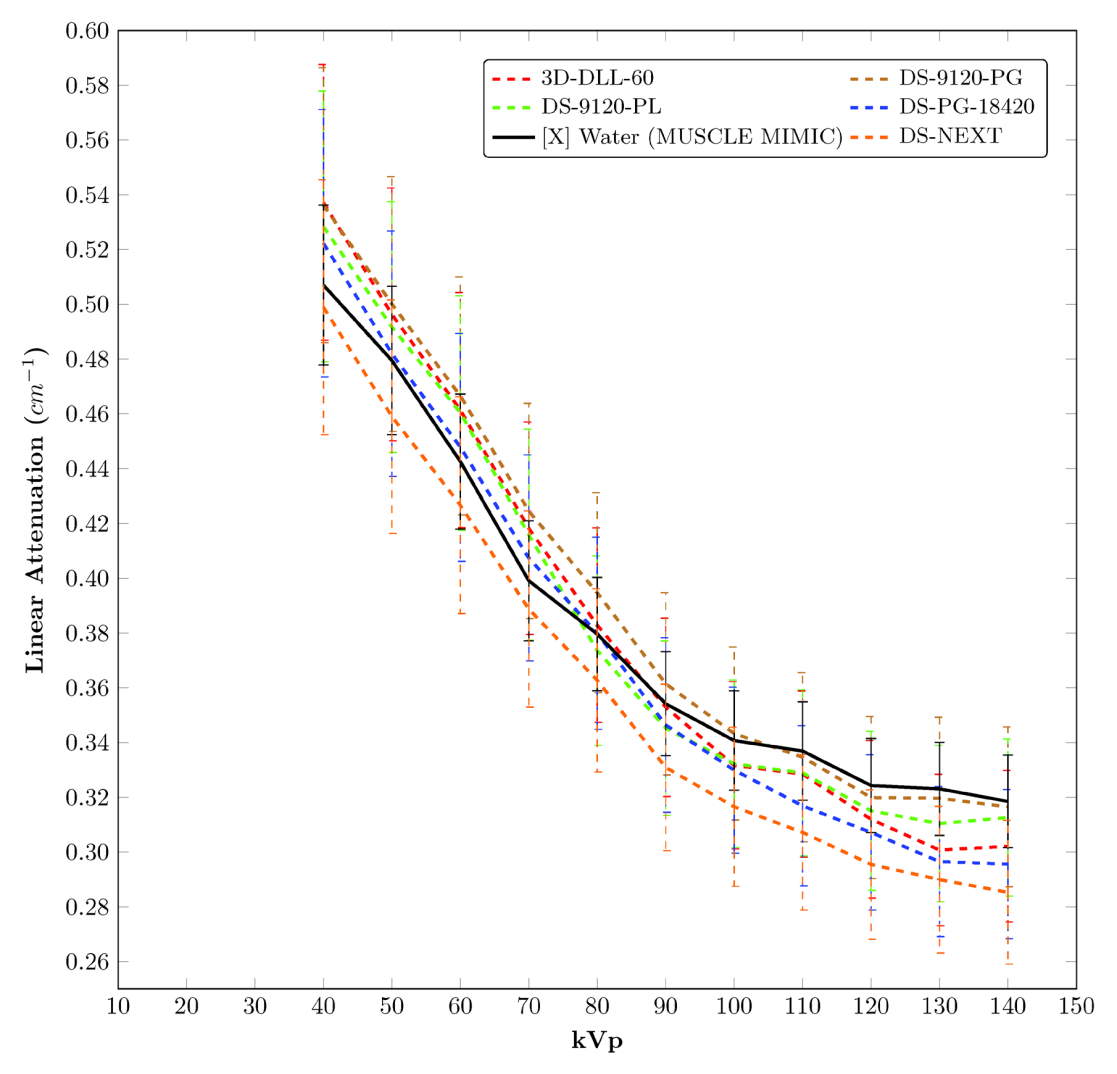
Candidate muscle mimics in this study (similar to water) (legend order is left to right and top to bottom). Average error relative to water: 3D-DLL-60 3.7%, DS-9120-PG 2.9%, DS-9120-PL 3.0%, DS-PG-18420 3.5%, and DS-NEXT 6.2%. Reference data from literature are identified with: [G] gamma source, [X] X-ray tube source, [C] calculated from atomic composition [27]. Data from this study have no preceding brackets.

The materials from this study with linear attenuation coefficients close to materials identified as polyenergetic (X-ray source) tissue mimics for muscle (water) are plotted in Fig. 4 [24].

**Fig. 5:**
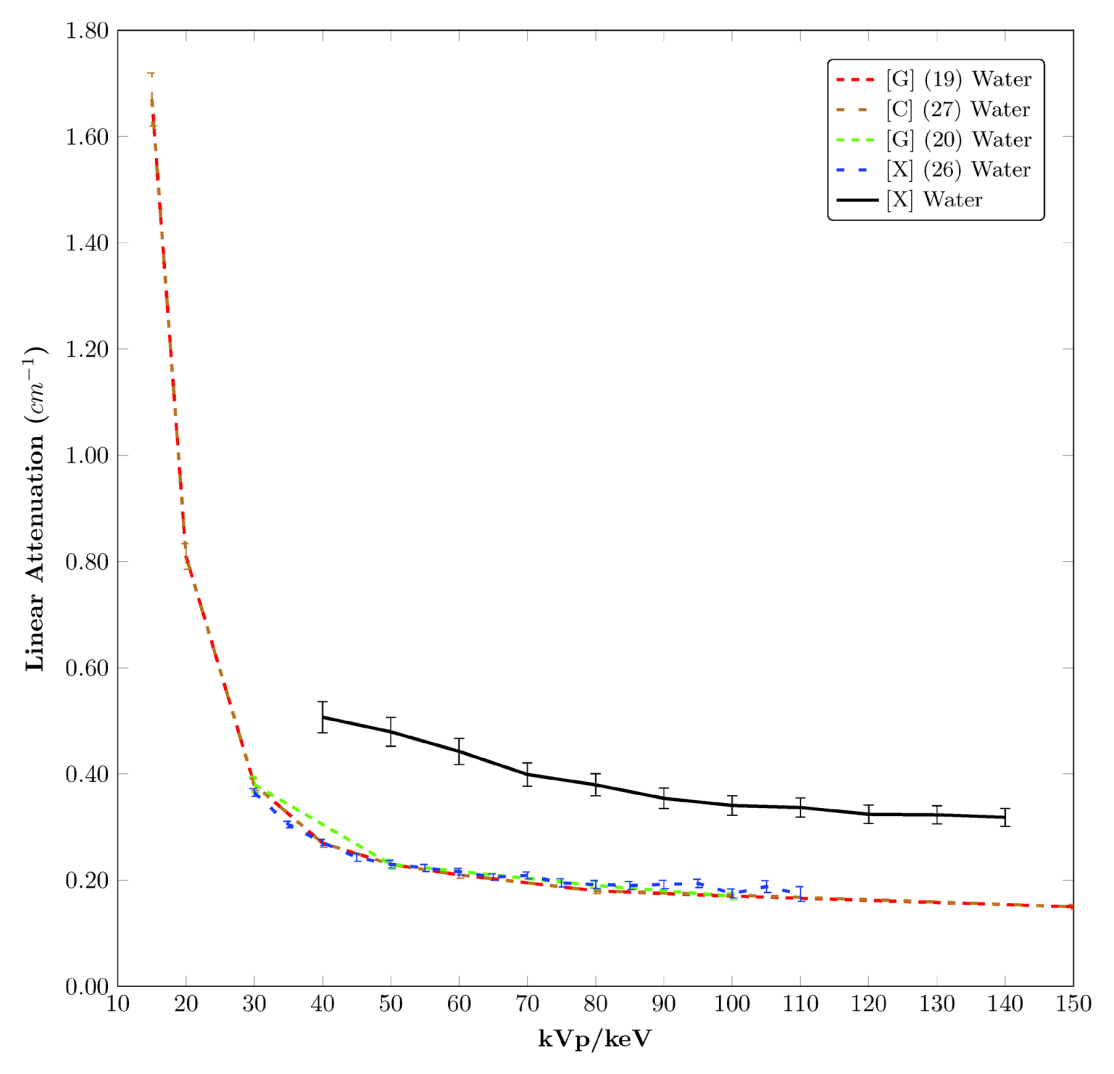
Comparison of water from this study with gamma source, X-ray source, and atomic calculations.

The water attenuation measured with the polyenergetic X-ray tube used in this study is compared to mono-energetic computational values and gamma sources in Fig. 5.

**Fig. 6:**
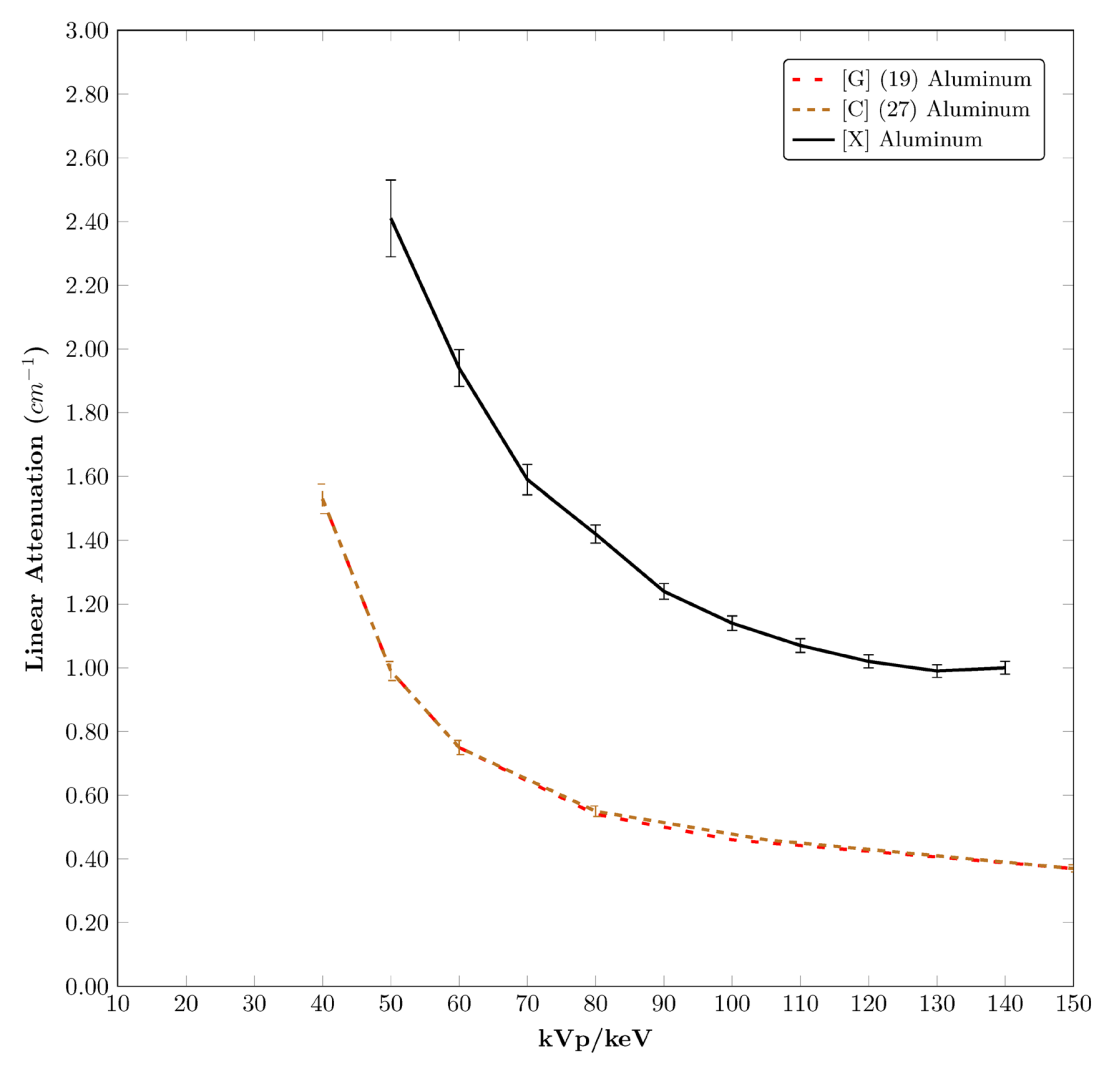
Comparison of aluminum coefficients from this X-ray study with gamma source and atomic calculation studies.

The attenuation of the aluminum sample measured for reference in this study is compared to literature reported values in Fig. 6.

## 4 Discussion

There are five polymers with linear attenuation curves within the error band of water. Twenty of the tested materials may be considered for fat mimics because their values fall in a range defined by PMMA attenuation at the top of the range and nylon (ST-NY-12PA) attenuation at the low end of the range [24]. The water attenuation measured with the polyenergetic X-ray tube used in this study plotted at higher values than the mono-energetic computational values and gamma sources due to the difference in source characteristics. The values for aluminum attenuation are higher than the values calculated for aluminum from atomic composition and gamma source measurements due to the polyenergetic characteristics of the X-ray tube used in this study. The wide range of density, elongation, tensile strength, and hardness values of the materials tested may be useful in mechanical phantoms; specifically, materials with Shore 00 hardness are very soft and are marketed as having skin-like texture, which would make them useful for anatomical modeling and mechanical testing. Polylactic acid (PLA) is a hydrophilic material that can be doped with chemical solutions to change the linear attenuation coefficient.

## 5 Conclusions

The X-ray linear attenuation coefficients of the tested material samples spanned a wide range of values, and small numbers of them overlapped established human tissue mimic values. Twenty 3D printer materials and one castable polyurethane tracked nylon and PMMA were established as X-ray mimics for fat. Five 3D printer materials tracked water as an established X-ray mimic for muscle.

## 6 Appendix A

**Table A1:** Linear attenuation coefficients reported in literature for tissues and mimics (cm^−1^).

		*Energy Incidence (keV)*										
Sample Name^a^	Density (kg/m^3^)	10	20	30	40	50	60	70	80	90	100	110
[C] (27) Lung inflated	260	1.42	0.22	0.10	0.07	0.06	0.05		0.05		0.04	
[G] (19) Polyethylene	930	1.94	0.40	0.21	0.18				0.17		0.16	
[G] (19) Water	1000	5.33	0.81	0.27	0.21				0.18		0.17	
[C] (21) Fat (est)			0.45	0.26	0.21	0.19	0.18	0.17	0.17		0.16	0.15
[X] (25) Fat	928		0.46	0.26	0.22	0.19			0.17			0.15
[X] (26) Polyethylene	948			0.26	0.22	0.20	0.19	0.19	0.17	0.18	0.18	0.16
[X] (26) Fat	920			0.28	0.22	0.19	0.19	0.18	0.17	0.16	0.17	0.17
[C] (27) Tissue adipose	916	3.00	0.52	0.28	0.22	0.19	0.18		0.16		0.15	
[C] (21) Liver (est)			0.75	0.36	0.26	0.22	0.20	0.19	0.18		0.17	0.16
[C] (27) Breast	1020	4.39	0.70	0.35	0.26	0.22	0.21		0.18		0.17	
[X] (26) Nylon	1138			0.33	0.26	0.24	0.23	0.23	0.21	0.21	0.19	0.23
[X] (26) Polycarbonate	1117			0.33	0.27	0.25	0.23	0.22	0.22	0.20	0.19	0.23
[C] (27) Water	1000	5.33	0.81	0.38	0.27	0.23	0.21		0.18		0.17	
[X] (26) Water	998.2			0.37	0.27	0.23	0.22	0.21	0.19	0.19	0.18	0.17
[X] (25) Breast fibrous	1035		0.80	0.38	0.27	0.23			0.19			0.17
[C] (21) Kidney (est)			0.83	0.38	0.27	0.23	0.21	0.20	0.19		0.18	0.17
[C] (27) Pancreas	1040	5.29	0.82	0.38	0.28	0.23	0.21		0.19		0.18	
[C] (21) Muscle (est)			0.85	0.39	0.28	0.23	0.21	0.20	0.19		0.18	0.17
[C] (27) Gastrointestinal tract	1042	5.39	0.83	0.39	0.28	0.23	0.21		0.19		0.18	
[C] (18) Muscle	1000		0.84	0.39	0.28	0.23	0.21		0.19		0.18	
[C] (27) Lymph	1030	5.62	0.85	0.39	0.28	0.23	0.21		0.19		0.18	
[C] (27) Cell nucleus	1000	6.05	0.91	0.40	0.28	0.23	0.21		0.15		0.17	
[X] (26) Kidney	1050			0.39	0.28	0.25	0.22	0.20	0.20	0.19	0.18	0.20
[C] (27) Muscle skeletal	1040	5.57	0.85	0.39	0.28	0.24	0.21		0.19		0.18	
[C] (27) Brain	1040	5.63	0.86	0.40	0.28	0.24	0.21		0.19		0.18	
[C] (27) Testis	1044	5.60	0.85	0.39	0.28	0.24	0.21		0.19		0.18	
[X] (25) Ductile carcinoma	1050		0.84	0.39	0.28	0.24			0.19			0.17
[C] (27) Ovary	1048	5.67	0.86	0.40	0.28	0.24	0.21		0.19		0.18	
[C] (27) Kidney	1050	5.63	0.55	0.39	0.28	0.24	0.22		0.16		0.18	
[C] (27) Liver	1053	5.69	0.87	0.40	0.28	0.24	0.22		0.19		0.18	
[C] (27) Eye lens	1100	5.31	0.83	0.39	0.28	0.24	0.22		0.20		0.18	
[C] (27) Spleen	1060	5.76	0.88	0.40	0.29	0.24	0.22		0.19		0.18	
[C] (27) Blood whole	1060	5.85	0.89	0.41	0.29	0.24	0.22		0.19		0.18	
[C] (27) Heart perfused	1060	5.81	0.89	0.41	0.29	0.24	0.22		0.19		0.18	
[C] (27) Skin	1100	5.45	0.84	0.40	0.29	0.25	0.22		0.20		0.19	
[X] (26) Liver	1045			0.41	0.30	0.26	0.23	0.23	0.21	0.20	0.20	0.17
[X] (26) Muscle	1040			0.41	0.30	0.26	0.24	0.24	0.22	0.22	0.22	0.24
[C] (27) Thyroid	1050	5.60	0.86	0.40	0.30	0.25	0.22		0.20		0.18	
[C] (27) Cartilage	1100	7.01	1.04	0.46	0.31	0.26	0.23		0.20		0.19	
[G] (19) Aluminum	2699	70.79	9.29	1.53	0.75				0.54		0.46	
[X] (26) Bone	1850			2.27	1.19	0.74	0.61	0.60	0.36	0.35	0.42	0.18
[C] (27) Bone cortical	1920	54.72	7.68	2.55	1.28	0.81	0.60		0.43		0.36	
[C] (27) Aluminum	2700	70.74	9.02	3.05	1.53	0.99	0.75		0.55		0.46	
[G] (20) Bone	1920			2.56		0.81			0.43		0.35	
[G] (20) Muscle	1044			0.40		0.24			0.19		0.18	
[G] (20) Water	997			0.38		0.23			0.19		0.17	

a Reference data from literature are identified with: [G] gamma source, [X] X-ray tube source, [C] calculated from atomic composition [27]

The linear attenuation coefficients reported in literature for tissues in Table A1 are plotted in Fig. A1.

**Table A2:** Physical characteristics and X-ray linear attenuation coefficient fit parameters for tested materials (*a*/*x^c^* + *b*, where *x* = kVp).

Print/ Cast^a^	Fabrication^a^	Sample Name^b^	Res^c^ (mm)	Density (kg/m^3^)	Elong^c^ (%)	TS^c^ (MPa)	Hard^c^ (SA)	*a*	*b*	*c*
Print	SLA	3D-Acc-25	0.102	1190	13.00	38.00	ND^d^	4.49	0.00	0.54
Print	SLA	3D-Acc-5530	0.051	1250	1.30	47.00	ND	5.10	0.00	0.56
Print	SLA	3D-Acc-60	0.051	1210	5.00	58.00	ND	3.53	0.00	0.51
Print	SLA	3D-Acc-XW200	0.102	1180	7.00	46.00	ND	5.75	0.14	0.82
Print	3DP	3D-CBZB	0.150	1040	20.00	ND	ND	65.72	0.06	0.97
Print	SLS	3D-Duraf	0.102	1200	4.50	31.00	ND	34.57	0.08	1.01
Print	SLS	AL-PA-614GS	0.102	1220	9.00	51.00	ND	36.10	0.11	1.01
Print	SLS	AL-PA650	0.102	1020	24.00	ND	ND	5.90	0.14	0.93
Print	SLS	AL-PA850	0.102	1030	51.00	48.00	ND	6.62	0.15	0.98
Ref	Ref	Aluminum	NA^d^	2699	12.00	124.00	ND	2488.03	0.70	1.86
Print	SLA	CN-CE220	0.150	1100	3.00	90.00	ND	4.71	0.12	0.75
Print	SLA	CN-PR25	0.150	1100	3.00	42.00	ND	3.08	0.04	0.57
Print	SLA	CN-RPU-70	0.150	1010	90.00	42.00	ND	2.49	0.06	0.57
Print	SLA	CR-EPU40	0.150	1000	190.00	6.00	68.00	1.84	0.00	0.46
Print	SLA	DS-9120-PG	0.051	1130	15.00	30.00	ND	4.50	0.09	0.63
Print	SLA	DS-9120-PL	0.051	1130	15.00	30.00	ND	3.09	0.00	0.47
Print	SLA	DS-NanoT	0.051	1650	0.70	66.30	ND	28.86	0.07	0.86
Print	SLA	DS-NanoT-V	0.051	1650	0.70	66.30	93.00	26.78	0.00	0.81
Print	SLA	DS-NEXT	0.102	1170	8.00	41.00	ND	3.06	0.01	0.49
Print	SLA	DS-PG-18420	0.102	1160	12.00	42.72	ND	3.29	0.00	0.49
Print	SLA	DS-WS-XC-11122	0.102	1120	11.00	47.00	ND	4.78	0.00	0.56
Print	SLS	ES-PA-1102B	0.150	990	45.00	48.00	ND	1.73	0.00	0.45
Print	SLS	ES-PA-2200	0.060	900	20.00	45.00	ND	2.23	0.04	0.55
Print	SLA	FL-BLK	0.025	1090	6.00	64.63	ND	4.47	0.10	0.71
Print	SLA	FL-CAST	0.025	1090	13.00	11.58	ND	5.40	0.12	0.79
Print	SLA	FL-CLR	0.025	1090	6.00	64.63	ND	3.81	0.08	0.67
Print	SLA	FL-DENT	0.050	1090	ND	ND	ND	3.24	0.06	0.60
Print	SLA	FL-DENTM	0.025	ND	5.00	61.00	ND	11.93	0.17	1.02
Print	SLA	FL-DUR	0.050	ND	67.00	31.80	ND	3.22	0.06	0.61
Print	SLA	FL-FLEX	0.050	1090	85.00	8.47	85.00	2.98	0.04	0.58
Print	SLA	FL-GREY	0.025	1090	6.00	64.63	ND	4.43	0.09	0.70
Print	SLA	FL-HITMP	0.025	1100	2.00	51.00	ND	3.42	0.06	0.62
Print	SLA	FL-TOUGH	0.050	1090	24.00	41.27	ND	2.94	0.02	0.54
Print	SLA	FL-WHT	0.050	1090	6.00	64.63	ND	3.27	0.03	0.57
Print	SLA	FN-MF-G	0.025	1170	6.10	44.90	ND	2.77	0.00	0.51
Cast	Poly	HN-RP-6400	NA	1040	251.00	7.88	52.00	3.86	0.08	0.68
Print	SLA	HN-RS-7820	0.051	1130	8.00	35.83	ND	4.65	0.00	0.61
Print	FDM	NT-PLA	0.2	1190	660.00	26.00	85(00)	19.66	0.10	1.35
Cast	Si (cond)	SI-GI-1000	NA	1090	300.00	3.62	30.00	39.27	0.05	0.99
Cast	Si (cond)	SI-GI-1040	NA	1100	225.00	3.62	35.00	50.26	0.09	1.05
Cast	Si (cond)	SI-GI-1110	NA	1080	450.00	1.89	6.00	40.71	0.06	1.00
Cast	Si (cond)	SI-GI-1110T	NA	1080	450.00	1.89	12.00	305.63	0.29	1.56
Cast	Si (cond)	SI-GI-1120	NA	1080	475.00	2.69	17.50	7030.67	0.38	2.38
Cast	Si (cond)	SI-GI-300	NA	1350	160.00	4.13	47.50	48.13	0.08	0.99
Cast	Si (cond)	SI-GI-311	NA	1140	150.00	2.24	43.00	40.56	0.05	0.97
Cast	Si (add)	SI-P-10	NA	1080	450.00	2.41	30.00	43.42	0.06	1.04
Cast	Si (add)	SI-P-15	NA	1080	460.00	3.27	41.00	36.91	0.03	0.96
Cast	Si (add)	SI-P-17	NA	1030	150.00	3.41	16.50	51.19	0.09	1.00
Cast	Si (add)	SI-P-20	NA	1080	425.00	3.62	50.00	43.17	0.06	1.01
Cast	Si (add)	SI-P-268	NA	1300	175.00	5.86	ND	488.83	0.35	1.64
Cast	Si (add)	SI-P-44	NA	1090	250.00	4.13	21.00	43.05	0.07	1.03
Cast	Si (add)	SI-P-45	NA	1120	275.00	5.51	42.00	47.16	0.08	1.04
Cast	Si (add)	SI-P-50	NA	1300	200.00	5.17	44.50	419.02	0.32	1.62
Cast	Si (add)	SI-P-60	NA	1240	200.00	6.20	53.00	19572.70	0.46	2.61
Cast	Si (add)	SI-P-90	NA	1130	415.00	4.13	59.00	7992.17	0.38	2.42
Cast	Si (add)	SI-XP-565	NA	1020	ND	ND	27.00	135.74	0.19	1.36
Cast	Si (add)	SI-XP-614	NA	1240	175.00	4.13	23.00	477.02	0.34	1.65
Cast	Si (add)	SI-XP-643	NA	1130	700.00	5.00	40.00	50.87	0.03	0.99
Cast	Si (add)	SI-XP-738	NA	1990	600.00	4.82	45.00	6829.39	0.36	2.39
Cast	Si (add)	SO-DS-10	NA	1070	1000.00	3.28	10.00	32.05	0.00	0.94
Cast	Si (add)	SO-DS-FX-PRO	NA	1062	763.00	1.98	2.00	35.98	0.02	0.98
Cast	Si (add)	SO-EF-00-20	NA	1070	845.00	1.10	20 (00)	27.60	0.01	0.94
Cast	Poly	SO-FF-ITI6	NA	900	ND	ND	ND	0.36	0.00	0.56
Cast	Si (add)	SO-SF15	NA	240	ND	ND	ND	11.63	0.02	1.08
Print	FDM	ST-ABS	0.254	1080	6.00	33.00	ND	480.61	0.19	2.10
Print	FDM	ST-ABS+P430-R	0.254	1040	6.00	33.00	ND	1.69	0.07	0.54
Print	FDM	ST-ABS+P430-U	0.178	1040	6.00	33.00	ND	2.73	0.09	0.69
Print	FDM	ST-ABS-ESD7	0.254	1040	3.00	36.00	ND	440.69	0.17	2.13
Print	FDM	ST-ABSi	0.254	1080	4.40	37.00	ND	2.71	0.08	0.63
Print	FDM	ST-ABS-M30	0.254	1040	7.00	32.00	NS	3.55	0.12	0.83
Print	FDM	ST-ABS-M30i	0.254	1040	4.00	36.00	ND	2.52	0.10	0.69
Print	PolyJet	ST-FLX2040	0.016	1120	110.00	1.30	37.50	939.37	0.22	2.24
Print	PolyJet	ST-FLX2050	0.016	1120	95.00	1.90	47.50	5.08	0.12	0.79
Print	PolyJet	ST-FLX2060	0.016	1120	75.00	2.50	60.00	3.59	0.09	0.66
Print	PolyJet	ST-FLX2070	0.016	1120	65.00	3.50	70.00	3.68	0.09	0.68
Print	PolyJet	ST-FLX2085	0.016	1120	55.00	5.50	82.50	5.68	0.13	0.82
Print	PolyJet	ST-FLX2095	0.016	1120	40.00	9.80	95.00	5.73	0.13	0.82
Print	PolyJet	ST-FLX930-P	0.038	1120	170.00	0.79	27.00	3.27	0.07	0.64
Print	PolyJet	ST-FLX930-U	0.016	1120	170.00	0.80	27.00	3.73	0.08	0.67
Print	PolyJet	ST-FLX9540	0.016	1120	100.00	1.30	40.00	5.63	0.12	0.83
Print	PolyJet	ST-FLX9550	0.016	1120	80.00	2.00	53.50	3.99	0.09	0.69
Print	PolyJet	ST-FLX9560	0.016	1120	60.00	2.80	63.50	4.30	0.10	0.74
Print	PolyJet	ST-FLX9570	0.016	1120	50.00	3.80	74.00	5.73	0.13	0.83
Print	PolyJet	ST-FLX9585	0.016	1120	35.00	6.00	86.00	6.11	0.13	0.84
Print	PolyJet	ST-FLX9595	0.016	1120	27.00	9.00	95.50	3.49	0.07	0.63
Print	FDM	ST-NY-12-PA	0.254	1160	30.00	46.00	ND	4.04	0.12	0.83
Print	FDM	ST-PC	0.254	1200	4.80	57.00	ND	3.89	0.09	0.72
Print	FDM	ST-PC-ABS	0.254	1098	5.00	34.00	ND	3.38	0.10	0.74
Print	PolyJet	ST-RGD5131DM	0.038	1170	25.00	55.00	ND	4.90	0.10	0.75
Print	PolyJet	ST-RGD5150	0.016	ND	18.00	45.00	ND	4.30	0.09	0.70
Print	PolyJet	ST-RGD525	0.016	1170	10.00	70.00	ND	5.68	0.11	0.81
Print	PolyJet	ST-RGD810	0.038	1180	10.00	50.00	ND	8.37	0.16	0.94
Print	PolyJet	ST-RGD835-PG	0.016	1170	10.00	50.00	NS^d^	5.67	0.13	0.81
Print	PolyJet	ST-RGD835-SC	0.038	ND	ND	ND	NS	3.91	0.10	0.68
Print	PolyJet	ST-RGD8455	0.016	ND	20.00	35.00	NS	4.13	0.09	0.69
Print	PolyJet	ST-RGD8460	0.016	ND	35.00	25.00	NS	1498.59	0.24	2.36
Print	Polyjet	ST-RGD875	0.016	1170	10.00	50.00	NS	3.21	0.07	0.61
Print	FDM	ST-UM 9085	0.254	1340	5.80	69.00	NS	621.88	0.23	2.10
Print	SLS	ST-VJ-PXL	0.102	1000	0.23	14.20	NS	56.22	0.00	0.91
Ref	Ref^d^	Water	NA	1000	NA	NA	NA	4.37	0.15	0.68

a (Print) printed, (Cast) cast, (SLA) stereolithography, (SLS) selective laser sintering, (FDM) fused deposition modeling, (3DP) 3D printing, (Si) silicone, (add) addition cure, (cond) condensation cure, (Poly) polyurethane. In silicone chemistry, a “condensation” cure uses a Tin (Sn) salt that expels water as a reaction by-product, whereas an “addition” cure uses a Platinum (Pt) reaction that creates an ethyl bridge between the polymer chains.

b The sample names are contractions of the manufacturer and the product name from the data sheet: (3D) 3D Systems, (AL) ALM, (CN) Carbon, (CR) Carbon Resin, (DS) DSM Somos, (ES) EOS, (FL) Formlabs, (FN) ProtoLabs FineLine, (NT) NinjaTek, (HN) Huntsman, (SI) Silicones, Inc., (SO) Smooth-On, Inc., (ST) Stratasys.

c (Res) resolution, (Elong) elongation, (TS) tensile strength, (Hard) hardness, (SA) Shore A, (00) Shore 00 scale (the two SO_EF-00-XX samples were very soft with skin-like hardness values measured on the Shore 00 scale for materials below the Shore A hardness scale).

d (ND) no data, (NA) not applicable, (NS) no signal, (Ref) reference.

The linear attenuation coefficients for the materials tested in Table A2 are plotted in Fig. A2.
